# Dreadnought Hospital

**Published:** 1887-12-10

**Authors:** P. Michelli

**Affiliations:** Secretary


### Dreadnought Seamen's Hospital.

In the list of hospitals, the representatives of which interested themselves in the effort that was made to draw public attention to the necessity of supporting hospitals through the medium of the Hospital Sunday Fund, you have omitted to mention this hospital. As every effort was made by the managers of this society to aid the movement, I trust you will be good enough to rectify what is apparently an error.

November 22nd, 1887. P. Michelli, Secretary.

[We much regret that this letter reached us too late for publication last week.—Ed.]

